# Updating the evidence on the effectiveness of the alcohol reduction app, Drink Less: using Bayes factors to analyse trial datasets supplemented with extended recruitment

**DOI:** 10.12688/f1000research.17952.2

**Published:** 2019-07-15

**Authors:** Claire Garnett, Susan Michie, Robert West, Jamie Brown

**Affiliations:** 1 Department of Behavioural Science and Health, University College London, London, UK; 2 Department of Clinical, Education and Health Psychology, University College London, London, UK

**Keywords:** Bayes Factors, digital interventions, alcohol reduction, smartphone apps

## Abstract

**Background**: A factorial experiment evaluating the Drink Less app found no clear evidence for main effects of enhanced versus minimal versions of five components but some evidence for an interaction effect. Bayes factors (BFs) showed the data to be insensitive. This study examined the use of BFs to update the evidence with further recruitment.

**Methods**: A between-subject factorial experiment evaluated the main and two-way interaction effects of enhanced versus minimal version of five components of Drink Less. Participants were excessive drinkers, aged 18+, and living in the UK. After the required sample size was reached (n=672), additional data were collected for five months. Outcome measures were change in past week alcohol consumption and Alcohol Use Disorders Identification Test (AUDIT) score at one-month follow-up, amongst responders only (those who completed the questionnaire). BFs (with a half-normal distribution) were calculated (BF<0.33 indicate evidence for null hypothesis; 0.33<BF<3 indicate data are insensitive).

**Results**: Of the sample of 2586, 342 (13.2%) responded to follow-up. Data were mainly insensitive but tended to support there being no large main effects of the enhanced version of individual components on consumption (0.22<BF<0.83) or AUDIT score (0.14<BF<0.98). Data no longer supported there being two-way interaction effects (0.31<BF<1.99). In an additional exploratory analysis, participants receiving four of the components averaged a numerically greater reduction in consumption than those not receiving any (21.6 versus 12.1 units), but the data were insensitive (BF=1.42).

**Conclusions**: Data from extended recruitment in a factorial experiment evaluating components of Drink Less remained insensitive but tended towards individual and pairs of components not having a large effect. In an exploratory analysis, there was weak, anecdotal evidence for a synergistic effect of four components. In the event of uncertain results, calculating BFs can be used to update the strength of evidence of a dataset supplemented with extended recruitment.

## Introduction

A factorial experiment evaluating the effect of ‘enhanced’ versus ‘minimal’ versions of five components of the alcohol reduction app, Drink Less, found no clear evidence for simple effects but did find evidence that two-way combinations of certain ‘enhanced’ components together resulted in greater reductions than ‘minimal’ versions ^1^. This was a planned analysis but should be interpreted with caution as the two-way interactive effects were not specifically hypothesised *a priori* and were part of multiple interactions tested. Findings of this sort are not uncommon in experimental studies. One approach is to start another randomised trial specifically to test this hypothesis. A potentially more efficient alternative is to extend the trial with further recruitment and test this and other hypotheses using Bayes factors ^2, 3^. We used this approach with the Drink Less app.

Bayes factors are a measure of strength of evidence and allow researchers to ‘top-up’ their results from one trial with additional data collected, regardless of the stopping rule, unlike frequentist statistics ^2^. The use of Bayes factors supports efficient, incremental model building ^3^, as evidence can be continuously accumulated until it is clear whether there is an association or not ^2, 4^. The rapid accumulation of large amounts of data about digital behaviour change interventions (DBCIs) offers the opportunity to apply emerging methods to their evaluation. DBCIs often have the capacity to continue automatic data collection beyond the end of a trial with little or no additional resources. This paper will illustrate how Bayes factors can be used to optimise a DBCI by updating evidence from an effectiveness trial using the example of Drink Less—an alcohol reduction app.

Bayes factors are the ratio of the average likelihood of two competing hypotheses being correct given a set of data and can overcome some of the issues associated with traditional frequentist statistics ^5^. They indicate the relevant strength of evidence for two hypotheses; when evaluating interventions, the two hypotheses are typically the alternative hypothesis (the intervention had the desired effect) and the null hypothesis (the intervention had no effect). Bayes factors, unlike frequentist statistics, can distinguish between two interpretations of a non-significant result: i) support for the null hypothesis of ‘no effect’ and ii) data are insensitive to detect an effect i.e. ‘unsure about the presence of an effect’ ^5, 6^. Calculating Bayes factors to supplement frequentist statistics is a quick and simple procedure with several software packages freely available (e.g. an online calculator developed by Zoltan Dienes ^7^). Researchers are actively encouraged to supplement, or even replace, classical frequentist hypothesis testing with a Bayesian approach to provide greater interpretative value to any non-significant results ^8^. This is important as often non-significant results are misinterpreted as evidence for no effect; a review of trials conducted in addictions research found that the reporting of ‘no difference’ was only appropriate in a small number of papers reporting this 9.

The use of Bayes factors also has another major advantage over the traditional frequentist approach that relates to the stopping rule. The traditional frequentist approach necessitates a strict stopping rule and a single analysis of data. Typically, this involves an *a priori* power calculation to specify the required sample size for data collection and the trial to end at that point. Subsequent ‘topping-up’ of existing data and re-analysing the new larger data set is ‘prohibited’ ^10^. This is because any *p*-value between 0 and 1 is equally likely if the null hypothesis is true, regardless of how much data are collected ^11^. Therefore, given enough time and data collection, a significant *p*-value will always be obtained even if the null hypothesis is true ^10^. So if researchers find a non-significant result—which cannot distinguish between support for the null hypothesis and being insensitive to detect an effect—then a new study would be required to build on these findings. Restarting the process is a waste of research resources but necessary in the context of using a frequentist approach for analysis because additional data collected cannot be analysed. However, this is not the case when using Bayes factors, as they are driven towards zero when the null hypothesis is true and additional data are collected ^10^. Therefore, researchers may use Bayes factors to analyse additional data to complement an employed stopping rule ^2^.

In the evaluation of DBCIs, using Bayes factors is beginning to complement traditional frequentist statistics ^4, 12^, and analysing additional data would be of particular benefit. Data collection for a DBCI effectiveness trial is typically automated and therefore does not require additional resources to continue after a pre-specified sample size is reached. Rapid evaluations of DBCIs and efficient accumulation of evidence can be used to inform future versions, keeping pace with advances in technology. Using Bayes factors to update findings about the relative plausibility of the two hypotheses allows researchers to assess the DBCI’s effectiveness in an ongoing manner ^4^. This remains useful when deciding about whether there is sufficient evidence to demonstrate effectiveness and, therefore, continued development ^13^. To the authors’ knowledge, no DBCIs have used additional data collected to supplement original effectiveness trial findings and no trials have used Bayes factors to provide further insight based on additional data. However, Bayes factors have been used in trials for superiority, non-inferiority and equivalence designs to allow for explicit quantification of evidence in favour of the null hypothesis ^14^. Bayesian analyses, more generally, are often used in clinical trials for dose finding, efficacy monitoring, toxicity monitoring, and for diagnosis/decision making ^15^. For example, Bayesian analyses were used to simultaneously monitor toxicity and efficacy in a parallel phase I/II clinical trial design for combination therapies ^16^.

DBCIs require novel methods of evaluation that are quick and timely to inform the optimisation of the intervention ^17^. The multiphase optimisation strategy (MOST) is a method for building, optimising and evaluating multicomponent behavioural interventions. It involves a series of steps identifying the set of intervention components to be examined and evaluating the effects of these components ^13, 18^. Factorial trial designs allow the simultaneous evaluation of the intervention components, which enables both the independent and interactive effects to be estimated ^13^. Using a factorial trial to evaluate a DBCI can overcome some of the challenges associated with using the traditional randomised controlled trial, such as prolonged duration from recruitment to publication and a high-cost trial implementation ^19, 20^. The results from a factorial trial can be used to make decisions about which components to retain when optimising the intervention ^18^.

The Drink Less smartphone app is a DBCI aimed at supporting people who drink excessively to reduce their alcohol consumption. It was developed using evidence and theory, following MOST. The app was analysed in a full factorial trial to assess the effectiveness of its five intervention modules and their effects on app usage and subsequent usability ratings ^21^. The stopping rule for data collection, in line with the frequentist approach to analysis, was pre-specified, although data collection continued under the same conditions as the original factorial trial. Analysis of the original trial data using Bayes factors indicated that the data were insensitive to detect main effects but that combinations of the modules appeared effective ^1^.

### Aims

The aims of this study are substantive and methodological:

1. To update the evidence on effectiveness of Drink Less app components singly and in combination. Specifically, what are the main and two-way interactive effects of the intervention modules on:
  i. Change in weekly alcohol consumption
  ii. Change in full Alcohol Use Disorders Identification Test (AUDIT) score
2 To demonstrate how Bayes factors can be used to analyse additional outcome data collected in effectiveness trials and update beliefs about hypotheses.

## Methods

### Design

A between-subject full factorial (2 ^5^) trial to evaluate the effectiveness of five intervention modules in the Drink Less app. The research questions were specified prior to the trial commencing and pre-registered on ISRCTN (registration number: ISRCTN40104069) and published in an open-access protocol paper ^21^.

### Participants

Participants were included in the study if they: were aged 18 or over; lived in the UK (only available on UK Apple app store and users had to select ‘UK’ for ‘Country?’); had an AUDIT score of 8 or above (indicative of excessive drinking ^22^); were interested in reducing their drinking (indicated by the question ‘why are you using this app?’ with users choosing ‘interested in drinking less’ over ‘just browsing’); provided an email address and had downloaded a ‘trial version’ of the app (described below).

The sample size for the original factorial trial was 672 providing 80% power (with alpha at 5%, 1:1 allocation and a two-tailed test) to detect a mean change in alcohol consumption of 5 units between the ‘enhanced’ and ‘minimal’ versions for each intervention module ^23^, comparable with a face-to-face brief intervention ^24^. This assumed a mean of 27 weekly units at follow-up in the control group, a mean of 22 units in the intervention group and a SD of 23 units for both (d=0.22).

Recruitment was undertaken via promotion from organisations, such as Public Health England, Cancer Research UK, and listing the app in the iTunes Store according to best practices for app store optimisation.

### Measures

Baseline measures included the AUDIT questionnaire and a socio-demographic assessment (age, gender, ethnic group, level of education, employment status and current smoking status). The primary outcome measure was self-reported change in past week alcohol consumption (the difference between one-month follow-up and baseline). Past week alcohol consumption was derived from the frequency (Q1) and quantity (Q2) questions of the AUDIT-Consumption (AUDIT-C) questionnaire. The secondary outcome measure was self-reported change in full AUDIT score; in addition to the three questions on consumption in the AUDIT-C, the full AUDIT includes questions assessing harmful alcohol use (e.g. alcohol-related injuries) and symptoms of dependence. Other secondary outcome measures included in the original, full factorial trial were usage data and usability ratings though were not considered in this paper. Details of these measures are described elsewhere ^1^, and the data and Bayes Factors calculated are reported on the Open Science Framework ( https://osf.io/kqm8b/).

### Interventions

The Drink Less app is a DBCI for people who drink excessively to help them reduce their alcohol consumption. It is freely available on the UK version of the Apple App Store for all smartphones and tablets running iOS8 or above. The content of the app did not change during the trial except for minor bug fixes (to ensure compatibility with iOS 10).

The app is structured around goal setting: users can set their own goals based on units, cost, alcohol free days or calories with information on the UK drinking guidelines, units and alcohol-related harms. There are five intervention modules that aim to help them achieve their goal: Normative Feedback (providing normative feedback on the user’s level of drinking relative to others); Cognitive Bias Re-training (a game to retrain approach-avoidance bias for alcoholic drinks); Self-monitoring and Feedback (providing a facility for self-monitoring of drinking and receipt of feedback); Action Planning (helping users to undertake action planning to avoid drinking), and Identity Change (promoting a change in identity in relation to alcohol). In the trial version of the app, the five intervention modules existed in two versions: i) an ‘enhanced’ version containing the predicted active ingredients and ii) a ‘minimal’ version that acted as a control.

A detailed description of the content, development and factorial trial evaluation of the app is reported in two separate papers ^1, 25^.

### Procedures

Data collection for the factorial trial began on 18 ^th^ May 2016 and the required sample of eligible users was reached on 10 ^th^ July 2016; follow-up data were collected until 28 ^th^ August 2016. Trial data was collected continuously for a further four months until 19 ^th^ December 2016 under the same conditions as the original factorial trial (i.e. a ‘trial version’).

Informed consent to participate in the trial was obtained from all participants on first opening the app. Users who consented to participate completed the AUDIT and a socio-demographic questionnaire, indicated their reason for using the app and provided their email address for follow-up (a prize of £100 was offered in an attempt to decrease the proportion of users leaving this field blank). Users were then provided with their AUDIT score and, those who met the inclusion criteria, were randomised to one of 32 experimental conditions using an automated algorithm within the app for block randomisation.

Follow-up was conducted 28 days after participants downloaded the app and the questionnaire consisted of the full AUDIT and usability measures. Follow-up was conducted in two ways: i) via email with a link to the questionnaire in an online survey tool (Qualtrics), which also sent up to four reminders, and ii) within the app. Participants included according to the original trial and stopping rule were due to complete the follow-up questionnaire up until 29 ^th^ August 2016 and were contacted via email (through Qualtrics) and the app. Participants due to complete the follow-up questionnaire from 30 ^th^ August onwards, were only contacted via the app.

### Ethical approval

Ethical approval for Drink Less from the UCL Ethics Committee under the ‘optimisation and implementation of interventions to change health-related behaviours’ project (CEHP/2013/508).

### Analysis

All analyses were conducted using R version 3.4.0. The analysis plan for this paper followed a similar analysis plan as for the original factorial trial (which was pre-registered on 13 ^th^ February 2016; ISRCTN40104069 ^21^).

Participant characteristics were reported descriptively by intervention module. A factorial between-subjects design was used to assess the main and two-way interactive effects of the five intervention modules on the primary and secondary outcome measures. Analyses were conducted amongst responders only, those who completed the follow-up questionnaire. Bayes factors were calculated for each analysis assessing the main and the two-way interaction effects of the five intervention modules on the outcome measures. The two-way interactions were defined as enhanced/enhanced versus minimal/minimal for each pair of intervention modules. The mean difference and standard error of the mean difference for each main and two-way interactive effect was calculated. A half normal distribution was used to specify the predicted effect. Peak at 0 (no effect) with a SD equal to the expected effect size. This is a conservative approach and represents a hypothesis that the intervention had a least some positive effect, with the effect being more likely to be smaller than larger. Bayes factors were calculated using an online calculator ^7^.

The expected effect size for the primary calculation of Bayes factors was a reduction of 5 units per week (d=0.22), reflecting a large effect and that of the power calculation for the original factorial trial. Bayes Factors were also calculated for a medium effect (reduction of 3 units per week), and a small effect (reduction of 0.5 units per week) to permit a relative judgment for screening purposes. The expected effect size for the secondary outcome measure was calculated by translating the estimated effect size for the primary outcome measure (d=0.22) into the equivalent mean difference score of 1.45 (mean=19.1, SD=6.56 [based on original trial users, n=672]). Bayes factors will be interpreted in terms of categories of evidential strength (see Table 1) ^5, 26^.

**Table 1.** Interpretation of Bayes factors.

Bayes factor	Interpretation
>30	Very strong evidence for H _1_
10–30	Strong evidence for H _1_
3–10	Moderate evidence for H _1_
1–3	Anecdotal evidence for H _1_
1	No evidence
0.33–1	Anecdotal evidence for H _0_
0.10–0.33	Moderate evidence for H _0_
0.03–0.10	Strong evidence for H _0_
<0.03	Very strong evidence for H _0_

H _1_, alternative hypothesis; H _0_, null hypothesis.

## Results

### Study sample

**Figure 1.**
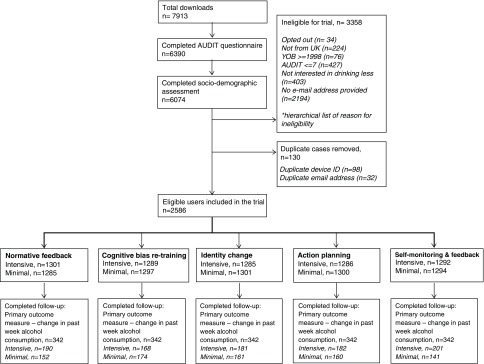
Flow chart of users.

The total sample size was 2586, of these 1914 (74.0%) were additional users to the original factorial trial (672, 26.0%). In total, 342 users (13.2%) completed the primary outcome measure in the follow-up questionnaire—the original users’ response rate was 26.6% and the additional users’ response rate was 8.5%. Figure 1 shows a flow chart of users throughout the study.

Socio-demographic and drinking characteristics of participants are reported in Table 2. Participants’ mean age was 37.2 years, 53.4% were women, 95.8% were white, 74.3% had post-16 qualifications, 87.0% were employed, and 30.0% were current smokers. Mean weekly alcohol consumption was 39.0 units, mean AUDIT-C score was 9.3, and mean AUDIT score was 19.1, indicating harmful drinking. Participants’ characteristics by intervention module are reported in Table 2. Generally, characteristics were similar for the enhanced and minimal version of each intervention module. The characteristics of participants who responded to the follow-up questionnaire (n=342) are reported in Supplementary Table 1.

**Table 2.** Participants’ characteristics at baseline. Data given as mean (SD), unless stated.

Variable	All	Normative Feedback		Cognitive Bias Re-training		Self-monitoring & Feedback		Action Planning		Identity Change	
		Enh	Min	Enh	Min	Enh	Min	Enh	Min	Enh	Min
Age	37.2 (10.64)	36.8 (10.49)	37.5 (10.77)	37.0 (10.56)	37.3 (10.72)	37.3 (10.88)	37.0 (10.38)	37.4 (10.87)	37.0 (10.40)	37.4 (10.75)	36.9 (10.52)
% Women (n)	53.4% (1381)	52.7% (686)	54.1% (695)	53.0% (693)	53.8% (688)	53.0% (685)	53.8% (696)	53.5% (688)	53.3% (693)	52.1% (670)	54.7% (711)
% White (n)	95.8% (2477)	96.1% (1250)	95.5% (1227)	96.3% (1241)	95.3% (1236)	95.7% (1237)	95.8% (1240)	95.8% (1232)	95.8% (1245)	95.3% (1224)	96.3% (1253)
% Post-16 qualifications (n)	74.3% (1921)	74.4% (968)	74.2% (953)	74.3% (958)	74.2% (963)	74.1% (958)	74.4% (963)	74.2% (954)	74.4% (967)	74.0% (951)	74.6% (970)
% Employed (n)	87.0% (2250)	87.0% (1132)	87.0% (1118)	87.1% (1123)	86.9% (1127)	85.6% (1106)	88.4% (1144)	85.1% (1095)	88.8% (1155)	86.6% (1113)	87.4% (1137)
% Current smokers (n)	30.0% (776)	29.7% (386)	30.4% (390)	29.8% (384)	30.2% (392)	28.9% (373)	31.1% (403)	29.8% (383)	30.2% (393)	28.0% (360)	32.0% (416)
Past week alcohol consumption (units)	39.0 (26.93)	39.0 (27.02)	39.0 (26.85)	39.7 (27.66)	38.3 (26.18)	39.2 (26.99)	38.8 (26.88)	38.8 (26.32)	39.2 (27.53)	38.7 (26.84)	39.3 (27.03)
AUDIT score	19.1 (6.66)	27.0 (19.18)	26.9 (18.98)	19.4 (6.78)	18.7 (6.52)	19.2 (6.63)	19.0 (6.69)	19.3 (6.67)	18.9 (6.64)	18.9 (6.56)	19.2 (6.76)
AUDIT-C score	9.3 (1.76)	9.3 (1.76)	9.3 (1.77)	9.3 (1.80)	9.3 (1.73)	9.3 (1.78)	9.3 (1.75)	9.3 (1.71)	9.3 (1.82)	9.3 (1.74)	9.3 (1.78)

Enh, enhanced; Min, minimal.

### Change in past week’s alcohol consumption

The main effects of the intervention modules are reported in Table 3 for the change in past week’s alcohol consumption. Bayes factors showed that the data were insensitive to detect an effect for *Normative Feedback* for effect sizes of 5-, 3- and 0.5-unit reductions (0.47<BF<0.97). Data were insensitive to detect an effect for *Cognitive Bias Re-training* for effect sizes of 5-, 3- and 0.5-unit reductions (0.74<BF<1.06). Bayes factors showed that the data were insensitive to detect an effect for *Self-monitoring and Feedback* for effect sizes of 5-, 3- and 0.5-unit reductions (0.43<BF<0.95). Bayes factors showed that the data were insensitive to detect an effect for *Action Planning* for effect sizes of 5-, 3- and 0.5-unit reductions (0.83<BF<1.08). Bayes factors for *Identity Change* showed support for the null hypothesis of no difference between the enhanced and minimal version of the module for a 5-unit reduction (BF=0.22), though data were insensitive to detect an effect for 3- and 0.5-unit reductions (0.34<BF<0.81). The data were insensitive to detect a two-way interactive effect between any pair of intervention modules for effect sizes of 5-, 3- or 0.5-unit reductions (0.35<BF<1.22), except for between *Self-monitoring and Feedback* and *Identity Change* for a 5-unit reduction which supported the null hypothesis (BF=0.31) (see Extended data, Supplementary Table 2 ^27^).

**Table 3.** Main effects of intervention modules on change in past week’s alcohol consumption. A negative number indicates a reduction over time.

Variable	Mean change in alcohol consumption, Units (SD)				Bayes factor		
	Enhanced	Minimal	F	P	B _H(0,5)_	B _H(0,3)_	B _H(0,0.5)_
Normative Feedback	-12.5 (25.70)	-12.7 (26.57)	0.007	0.933	0.47	0.66	0.97
Cognitive Bias Re-training	-13.4 (26.93)	-11.9 (25.22)	0.280	0.597	0.74	0.96	1.06
Self-monitoring and Feedback	-12.3 (24.97)	-13.0 (27.61)	0.052	0.820	0.43	0.62	0.95
Action Planning	-13.5 (24.70)	-11.6 (27.55)	0.443	0.506	0.83	1.06	1.08
Identity Change	-10.7 (27.76)	-14.8 (23.89)	2.144	0.144	0.22	0.34	0.81

### Change in AUDIT score

The main effects of the intervention modules are reported in Table 4 for the change in AUDIT score. The data were insensitive to detect an effect on change in AUDIT score for: *Normative Feedback* (BF=0.60); *Cognitive Bias Re-training* (BF=0.98); and *Action Planning* (BF=0.95). The data supported evidence for the null hypothesis of no difference in AUDIT score between enhanced and minimal versions of *Self-monitoring and Feedback* (BF=0.15) and *Identity Change* (BF=0.14). The two-way interactive effects of intervention modules on change in AUDIT score (see Extended data, Supplementary Table 3 ^27^) showed that the majority of data were insensitive to detect any two-way interactive effects (0.33<BF<1.99). Data supported the null hypothesis for no difference between enhanced and minimal versions between *Normative Feedback* and *Identity Change* (BF=0.29) and *Self-Monitoring and Feedbac*k and *Identity Change* (BF=0.18).

**Table 4.** Main effects of intervention modules on change in AUDIT score.

Variable	Mean change in AUDIT score (SD)				Bayes factor
	Enhanced	Minimal	F	P	B _H(0,1.45)_
Normative Feedback	-2.4 (5.55)	-2.04 (6.11)	0.298	0.586	0.60
Cognitive Bias Re-training	-2.5 (5.73)	-1.9 (5.88)	1.042	0.308	0.98
Self-monitoring and Feedback	-1.8 (5.62)	-2.8 (6.02)	3.006	0.084	0.15
Action Planning	-2.5 (6.06)	-1.9 (5.50)	0.983	0.322	0.95
Identity Change	-1.7 (5.89)	-2.8 (5.66)	3.529	0.061	0.14

### Exploratory analysis of a synergistic effect on change in past week’s alcohol consumption

Four intervention modules ( *Normative Feedback*, *Cognitive Bias Re-Training*, *Self-Monitoring and Feedback*, and *Action Planning*) have some evidence in support of their role of reducing alcohol consumption. Therefore, an additional exploratory analysis was conducted to assess whether there is a larger cumulative effect of the combination of all four modules in the enhanced version compared with the minimal version. This was done for responders only (n=39; 12 “off” vs 27 “on”) and for last observation carried forward (n=324; 164 “off” vs 160 “on”) to provide potential evidence for what effect size we can expect when planning a definitive trial with longer-term follow-up. Last observation carried forward means that participants’ past week alcohol consumption at follow-up was used for all of those who responded to follow-up and the baseline measure for past week alcohol consumption was used for those who did not respond to follow-up. Whilst last observation carried forward has its limitations, it maintains the variability within the data. Table 5 reports the Bayes factors for these analyses. There was a large numerical difference between all enhanced and all minimal for the four modules amongst responders only, although the Bayes factors found that the data were insensitive to detect an effect, which may be due in part to the small sample size.

**Table 5.** Four modules in ‘enhanced’ vs four modules in ‘minimal’ versions for past week alcohol consumption.

Variable	PWAC, Units (SD)				Bayes factor		
	All enhanced	All minimal	F	P	B _H(0,5)_	B _H(0,3)_	B _H(0,0.5)_
Responders only (change in PWAC)	-21.6 (20.36)	-12.1 (26.82)	1.474	0.232	1.42	1.29	1.05
Last observation carried forward (PWAC)	36.7 (28.48)	37.4 (26.59)	0.059	0.808	0.62	0.82	1.02

PWAC, past week alcohol consumption.

## Discussion

The calculation of Bayes factors for additional data collected beyond the original factorial trial of Drink Less has allowed us to accumulate and update existing evidence on the effectiveness of its intervention components in reducing alcohol consumption. The supplemented data remained insensitive to detect whether the Drink Less app components have large (5-unit) individual or two-way interactive effects on reducing alcohol consumption though tended towards anecdotal evidence for the null hypothesis of no effect. There was evidence of two-way interactive effects in the original factorial trial that is no longer supported by the supplemented data.

The current data also remained insensitive to detect whether the four most promising components ( *Normative Feedback, Cognitive Bias Re-Training*, *Self-Monitoring and Feedback* and *Action Planning*) may each have effects smaller than 5 units. An unplanned analysis provided weak anecdotal evidence of a synergistic effect of the ‘enhanced’ versions of these four intervention modules together. On both past week alcohol consumption and AUDIT score, and across several alternative effect sizes, there was support for no effect of the fifth intervention module, *Identity Change*. These findings, alongside results from analysing user feedback and usage data on the most frequently visited screens, guided the decision to remove the *Identity Change* module from the next major app update whilst retaining *Normative Feedback* and *Cognitive Bias Re-Training*, and *Self-Monitoring and Feedback* and *Action Planning*.

Whilst this study did not find evidence of a large individual effect of any of the intervention modules, there remains some evidence to suggest that an optimised version of the app (with the removal of the *Identity Change* module) may yet prove effective. As with the original factorial trial, there are concerns that the minimal versions were too active in an attempt to promote engagement amongst all participants. Even participants who were randomised to receive the minimal versions of every intervention module were able to set goals and track their drinks, which is associated with reduced consumption ^28^. Most alcohol reduction apps include few techniques to change behaviour ^29^ suggesting that even the minimal version of Drink Less was more active than most existing alcohol reduction apps. Therefore, effectiveness estimates derived from this approach are likely to be conservative. Furthermore, Drink Less users have excellent levels of engagement with the app ^30^, which is necessary (but not sufficient) for an intervention to be effective. Additionally, a content analysis of user feedback (available as a short report here: https://osf.io/d3w8r/) found that of the ‘Information giving’ category, the majority provided positive feedback on the app as a whole. A sample of the user feedback is available to view on the Drink Less website ^31^. Drink Less is also one of the leading alcohol reduction apps in the UK with over 50,000 unique users and an average 4.1-star rating (as of June 2019).

A major strength of this study is its illustration of how it is possible to evaluate data from trials of DBCIs in an on-going manner. No additional resources were required to continue data collection within the original trial of Drink Less as the app remained freely available on the UK Apple app store and the notification to complete the follow-up questionnaire had already been programmed. Analysing the supplemented dataset has allowed us to update our findings and provided more confidence in our original decisions on which components to retain or remove as part of the process of optimising the intervention ^18^ to improve its effectiveness and usability. We are also much clearer that any definitive trial must be powered to detect small effects and designed to inform a pragmatic decision about whether to invest resources in recommending the app. The optimisation of the Drink Less intervention was based on the findings from this study as well as on user feedback and findings from a meta-analysis of the intervention components in digital alcohol interventions associated with effectiveness ^32^. The findings from this study informed the removal of the ‘Identity Change’ module and retention of the remaining four modules.” The stopping rule in frequentist statistics means that additional trial data collected as part of an effectiveness trial for a DBCI would go to waste. The use of Bayes factors in this situation prevents unnecessary waste of resources and enables researchers to continually update their evidence on a DBCI rather than collect and analyse individual data sets as part of separate trials.

A limitation of this study and the use of Bayes factors was that we were not able to use the intention-to-treat (ITT) approach in the analysis (as was done for the original trial), whereby those lost to follow-up (non-responders) were assumed to be drinking at baseline levels. Whilst Bayes factors can overcome a lot of the issues with the frequentist approach, they are not meaningful when assumptions are made that limit the variability in the data. Due to low overall follow-up rates (13.2%) in this larger sample, the ITT assumption that there was no change in the large majority of the sample drives the variability down, which in turn drives support for the null hypothesis. This highlights that Bayes factors were not useful in this study when using the ITT assumption, which limits the variability in the data.

We acknowledge that the follow-up rate is very low and this is likely to be due to the lack of financial incentive for completing the follow-up survey, which are known to increase response rates in randomised trials ^33^. Furthermore, the follow-up rate in the extended dataset was lower than for the original trial dataset; this is likely because participants were only contacted via the app for the extended dataset whilst the participants in the original dataset were also contacted via email.

The intervention modules of the Drink Less app do not have a large individual effect on reducing alcohol-related outcomes, though they may have a small effect that the current data were unable to detect. There is weak evidence for a synergistic effect of the ‘enhanced’ versions of four intervention modules together: *Normative Feedback* and *Cognitive Bias Re-Training*, and *Self-Monitoring and Feedback* and *Action Planning*. This study has updated the existing evidence on the effectiveness of intervention modules in the Drink Less app. In the event of uncertain results following a primary analysis, Bayes factors can be used to ‘top-up’ results from DBCI trials with any additional data collected, therefore supporting efficient, incremental model building to inform decision-making.

## Data availability

### Underlying data

A dataset containing the extended trial outcomes is available on OSF. DOI: https://doi.org/10.17605/OSF.IO/KQM8B ^27^.

### Extended data

Extended data are available on OSF. DOI: https://doi.org/10.17605/OSF.IO/KQM8B ^27^.

Supplementary Table 1. Participants’ characteristics (who responded to follow-up, n=342) at baseline.

Supplementary Table 2. Two-way interactive effects of intervention modules on change in past week’s alcohol consumption.

Supplementary Table 3. Two-way interactive effects of intervention modules on change in AUDIT score.

Data are available under the terms of the Creative Commons Zero "No rights reserved" data waiver (CC0 1.0 Public domain dedication).
