# Supplementary material for: Updating the evidence on the effectiveness of the alcohol reduction app, Drink Less: using Bayes factors to analyse trial datasets supplemented with extended recruitment
Source: F1000Res. 2019 Jul 15;8:114. Originally published 2019 Jan 29. [Version 2] doi: 10.12688/f1000research.17952.2 (PMC6635983; doi:10.12688/f1000research.17952.2)
Supplement: Peer review history

# Peer review: Reviewer response for version 2

**DOI:** 10.5256/f1000research.21823.r51200

**Published:** 2019-07-16

**Authors:** Nick Heather

**Affiliations:** 1 Department of Psychology, Faculty of Health & Life Sciences, Northumbria University, Newcastle upon Tyne, UK

The authors have responded satisfactorily to criticisms of the previous version of this paper. I have no further comments to make.

I confirm that I have read this submission and believe that I have an appropriate level of expertise to confirm that it is of an acceptable scientific standard.

# Peer review: Reviewer response for version 2

**DOI:** 10.5256/f1000research.21823.r51199

**Published:** 2019-07-16

**Authors:** Daniel Leightley

**Affiliations:** 1 King's Centre for Military Health Research, Institute of Psychiatry, Psychology & Neuroscience, King's College London, London, UK

The authors have suitably addressed my concerns. I have no outstanding issues that need to be addressed.

I confirm that I have read this submission and believe that I have an appropriate level of expertise to confirm that it is of an acceptable scientific standard.

# Peer review: Reviewer response for version 1

**DOI:** 10.5256/f1000research.19633.r44925

**Published:** 2019-03-13

**Authors:** Nick Heather

**Affiliations:** 1 Department of Psychology, Faculty of Health & Life Sciences, Northumbria University, Newcastle upon Tyne, UK

This paper reports the results of a factorial experiment evaluating the Drink Less Smart phone app and its behaviour change components, with the benefit of an extended data-set. The aims of the study were both substantive and methodological. In relation to the latter, the paper provides a very useful example of the advantages of Bayesian hypothesis testing and in particular its legitimate provision for extending data collection until a firm conclusion has been reached. The paper also provides a good example, with earlier papers, of the MOST method for developing and evaluating behaviour change interventions.

 Regarding the substantive aim, I have two major and several minor comments.

**MAJOR:**

1. Analysis of the full data-set after extension confirmed the mainly inconclusive results of tests of the 5 individual components of the app in the original evaluation study and failed to confirm the previously reported evidence in favour of two interaction effects between components. In view of the authors' thorough and painstaking development of the app over the years, their rigorous evaluations of the components of the app and their stated intention to optimise the app for a definitive test in an RCT with extended follow-up, these results must be regarded as very disappointing. Yet this is not commented on and no possible explanation is offered why components whose inclusion was supported strongly by theory and previous research failed to show effects on drinking behaviour. Is it possible, as the authors have previously suggested, that the 'minimal', control interventions were too active to allow an effect to emerge? What other explanations are there for these disappointing results? Overall, where do the authors go from here in the attempt to bring this very promising intervention technology to practical use?
2. In the Abstract the authors state: 'There was weak evidence for a synergistic effect of four components'. I feel even this is too strong. In the text on p.9 we have: *'*An unplanned analysis provided weak anecdotal evidence of a synergistic effect of the ‘enhanced’ versions of these four intervention modules together'. Something along these lines, with the inclusion of 'unplanned' and 'anecdotal' would be more appropriate for the Abstract.

Another concern here is that the putative effect in question derives from a post hoc hypothesis based on unplanned comparisons arrived at only after the extension of data collection. In a frequentist approach to hypothesis testing, this would not of course be permissible but, even in the Bayesian approach, there must surely be some constraints on the legitimacy of testing post hoc hypotheses derived from exploratory analyses (rather than using such analyses to generate hypotheses not tested in the present data). The authors should comments on this issue and, if necessary, seek expert advice.

**MINOR:**

1. Why was the follow-up rate so much lower in the extended data-set that in the original data (8.5% versus 26.6%)? Can the authors attempt to explain this difference?
2. p.8: '... to provide potential evidence for what effect size we can expect when planning the trial.' This is presumably the definitive trial with extended follow-up but this is not clear.
3. What about the data on secondary outcomes? These are not reported here but it is not stated that they will not be considered in this paper and the reader is not told where they will be found.
4. The primary outcome measure  of self-reported change in past week alcohol consumption was presumably based on the AUDIT-C questionnaire, as suggested by Table 2, but this is not made clear in the text.

I confirm that I have read this submission and believe that I have an appropriate level of expertise to confirm that it is of an acceptable scientific standard, however I have significant reservations, as outlined above.

# Response

**Published:** 2019-07-04

**Authors:** Claire Garnett

**Affiliations:** University College London, UK

Analysis of the full data-set after extension confirmed the mainly inconclusive results of tests of the 5 individual components of the app in the original evaluation study and failed to confirm the previously reported evidence in favour of two interaction effects between components. In view of the authors' thorough and painstaking development of the app over the years, their rigorous evaluations of the components of the app and their stated intention to optimise the app for a definitive test in an RCT with extended follow-up, these results must be regarded as very disappointing. Yet this is not commented on and no possible explanation is offered why components whose inclusion was supported strongly by theory and previous research failed to show effects on drinking behaviour. Is it possible, as the authors have previously suggested, that the 'minimal', control interventions were too active to allow an effect to emerge? What other explanations are there for these disappointing results? Overall, where do the authors go from here in the attempt to bring this very promising intervention technology to practical use?

*This is a very good point made by both reviewers that we do not sufficiently discuss the value proposition of Drink Less in our discussion section. We have added a paragraph on this:*

*“Whilst this study did not find evidence of a large individual effect of any of the intervention modules, there remains some evidence to suggest that an optimised version of the app (with the removal of the ‘Identity Change’ module) may yet prove effective. As with the original factorial trial, there are concerns that the minimal versions were too active in an attempt to promote engagement amongst all participants. Even participants who were randomised to receive the minimal versions of every intervention module were able to set goals and track their drinks, which is associated with reduced consumption *[1] *. Most alcohol reduction apps include few techniques to change behaviour *[2] *suggesting that even the minimal version of Drink Less was more active than most existing alcohol reduction apps. Therefore, effectiveness estimates derived from this approach are likely to be conservative. Furthermore, Drink Less users have excellent levels of engagement with the app *[3] *, which is necessary (but not sufficient) for an intervention to be effective. Additionally, a content analysis of user feedback (available as a short report here:* https://osf.io/d3w8r/ *) found that of the ‘Information giving’ category, the majority provided positive feedback on the app as a whole. A sample of the user feedback is available to view on the Drink Less website *[4] *. Drink Less is also one of the leading alcohol reduction apps in the UK with over 50,000 unique users to date with an average 4.1-star rating (as of June 2019).”*

In the Abstract the authors state: 'There was weak evidence for a synergistic effect of four components'. I feel even this is too strong. In the text on p.9 we have: *'*An unplanned analysis provided weak anecdotal evidence of a synergistic effect of the ‘enhanced’ versions of these four intervention modules together'. Something along these lines, with the inclusion of 'unplanned' and 'anecdotal' would be more appropriate for the Abstract.

*We have re-worded the abstract to be cautious with our conclusions:*

*“In an additional exploratory analysis, participants receiving four of the components averaged a numerically greater reduction in consumption than those not receiving any (21.6 versus 12.1 units), but the data were insensitive (BF=1.42).*

*Data from extended recruitment in a factorial experiment evaluating components of the Drink Less app remained insensitive but tended towards individual and pairs of components not having a large effect. In an exploratory analysis, there was weak anecdotal evidence for a synergistic effect of four components. In the event of uncertain results, calculating BFs can be used to update the strength of evidence of a dataset supplemented with extended recruitment.”*

Another concern here is that the putative effect in question derives from a post hoc hypothesis based on unplanned comparisons arrived at only after the extension of data collection. In a frequentist approach to hypothesis testing, this would not of course be permissible but, even in the Bayesian approach, there must surely be some constraints on the legitimacy of testing post hoc hypotheses derived from exploratory analyses (rather than using such analyses to generate hypotheses not tested in the present data). The authors should comments on this issue and, if necessary, seek expert advice.

*These analyses were exploratory rather than testing a post-hoc hypothesis and the Bayesian approach does not have the same limitations as the frequentist approach in conducting exploratory analyses. Also, the conclusions match the strength of the evidence for this exploratory analysis (weak and anecdotal). We plan to pre-register any hypotheses and analysis plans in future studies. Furthermore, a key part of this study was for it to be informative in making decisions on which components to retain in an optimised version of the app and in terms of conducting a power analysis for a definitive trial.*

Why was the follow-up rate so much lower in the extended data-set that in the original data (8.5% versus 26.6%)? Can the authors attempt to explain this difference?

*We have included a discussion of this in the limitations:*

*“We acknowledge that the follow-up rate is very low and this is likely to be due to the lack of financial incentive for completing the follow-up survey, which are known to increase response rates in randomised trials (Brueton et al., 2014). Furthermore, the follow-up rate in the extended dataset was lower than for the original trial dataset; this is likely because participants were only contacted via the app for the extended dataset whilst the participants in the original dataset were also contacted via email.”*

p.8: '... to provide potential evidence for what effect size we can expect when planning the trial.' This is presumably the definitive trial with extended follow-up but this is not clear.

*We have clarified this:*

*“…to provide potential evidence for what effect size we can expect when planning a definitive trial with longer-term follow-up.”*

What about the data on secondary outcomes? These are not reported here but it is not stated that they will not be considered in this paper and the reader is not told where they will be found.

*The data on the other secondary outcomes (usage data and usability ratings) were not reported in this paper though we have added information on where they will be found:*

*“Other secondary outcome measures included in the original, full factorial trial were usage data and usability ratings though were not considered in this paper. Details of these measures are described elsewhere 1, and the data and Bayes Factors calculated are reported on the Open Science Framework (https://osf.io/kqm8b/).”*

The primary outcome measure of self-reported change in past week alcohol consumption was presumably based on the AUDIT-C questionnaire, as suggested by Table 2, but this is not made clear in the text.

*We have clarified this in the measures section:*

*“The primary outcome measure was self-reported change in past week alcohol consumption (the difference between one-month follow-up and baseline). Past week alcohol consumption was derived from the frequency (Q1) and quantity (Q2) questions of the AUDIT-Consumption (AUDIT-C) questionnaire.”*

# Peer review: Reviewer response for version 1

**DOI:** 10.5256/f1000research.19633.r43750

**Published:** 2019-02-11

**Authors:** Daniel Leightley

**Affiliations:** 1 King's Centre for Military Health Research, Institute of Psychiatry, Psychology & Neuroscience, King's College London, London, UK

This paper describes the effectiveness of DrinkLess, a mobile alcohol intervention, using Bayes factor to further compliment previously published findings. I have one major comment and several minor comments.

 Major Comment:

1. The work extends our understanding of DrinkLess and its effectiveness in managing alcohol misuse; however, it would be helpful to make a clear statement on how the Bayes analysis has improved the value proposition of DrinkLess.

Minor Comments:

1. Abstract: “Amongst responders only” – is that the sample who took part in the follow-up questionnaire?;
2. Abstract: “Unplanned comparison” appears to convey a negative connotation the authors could alter to “additional analyses”;
3. Abstract: “four most promising” could be misleading as you only had five components but we also have to be mindful that the data was insensitive;
4. Abstract: “reminded insensitive but tended” are you able to provide any BF for this statement?;
5. Introduction: It would be helpful to provide some discussion (specific examples) on how Bayes have been used in other domains to provide more insight by means of additional data;
6. Methods – Participants: “were interested in reducing their drinking” how was this measured? Were participants research aware, or were they targeted because they had previously stated an interest in reducing alcohol? Or could it be by downloading DrinkLess they were assumed to be interested in reducing their alcohol consumption?;
7. Methods – Participants: Was a geolocation restriction placed on participants? How can you be sure that users were from the UK?;
8. Methods – Intervention: What were the minor bug fixes, is a summary able to be provided as a supplement?;
9. Results: Results present AUDIT-C score, however this is not discussed previously.
10. Results: It would be helpful to have Table 2 represented as supplementary material for those who took part in follow-up;
11. Results: It would be of interest to discuss further the difference in AUDIT and AUDIT-C score and the role the final two questions (risk taking etc) play;
12. Discussion: “no additional resources were required” – is this the case, was the app provisioned for longer than anticipated?;
13. Discussion: “our decision on which components to retain or remove” – a bit more discussion round this aspect would be helpful to the reader;
14. Discussion: A 13.2% follow-up rate appears to be very low, do the authors have any reasons for this?;

I confirm that I have read this submission and believe that I have an appropriate level of expertise to confirm that it is of an acceptable scientific standard, however I have significant reservations, as outlined above.

# Response

**Published:** 2019-07-04

**Authors:** Claire Garnett

**Affiliations:** University College London, UK

The work extends our understanding of DrinkLess and its effectiveness in managing alcohol misuse; however, it would be helpful to make a clear statement on how the Bayes analysis has improved the value proposition of DrinkLess.

*This is a very good point made by both reviewers that we do not sufficiently discuss the value proposition of Drink Less in our discussion section. We have added a paragraph on this:*

*“Whilst this study did not find evidence of a large individual effect of any of the intervention modules, there remains some evidence to suggest that an optimised version of the app (with the removal of the ‘Identity Change’ module) may yet prove effective. As with the original factorial trial, there are concerns that the minimal versions were too active in an attempt to promote engagement amongst all participants. Even participants who were randomised to receive the minimal versions of every intervention module were able to set goals and track their drinks, which is associated with reduced consumption *[1] *. Most alcohol reduction apps include few techniques to change behaviour *[2] *suggesting that even the minimal version of Drink Less was more active than most existing alcohol reduction apps. Therefore, effectiveness estimates derived from this approach are likely to be conservative. Furthermore, Drink Less users have excellent levels of engagement with the app *[3] *, which is necessary (but not sufficient) for an intervention to be effective. Additionally, a content analysis of user feedback (available as a short report here:* https://osf.io/d3w8r/ *) found that of the ‘Information giving’ category, the majority provided positive feedback on the app as a whole. A sample of the user feedback is available to view on the Drink Less website *[4] *. Drink Less is also one of the leading alcohol reduction apps in the UK with over 50,000 unique users to date with an average 4.1-star rating (as of June 2019).”*

Abstract: “Amongst responders only” – is that the sample who took part in the follow-up questionnaire?;

*Yes, this has been clarified in the abstract with “…amongst responders only (those who completed the questionnaire).”*

Abstract: “Unplanned comparison” appears to convey a negative connotation the authors could alter to “additional analyses”;

*We have reworded this to “additional exploratory analysis” throughout the manuscript.*

Abstract: “four most promising” could be misleading as you only had five components but we also have to be mindful that the data was insensitive;

*We have re-worded the abstract to be cautious with our conclusions:*

*“In an additional exploratory analysis, participants receiving four of the components averaged a numerically greater reduction in consumption than those not receiving any (21.6 versus 12.1 units), but the data were insensitive (BF=1.42).*

*Data from extended recruitment in a factorial experiment evaluating components of the Drink Less app remained insensitive but tended towards individual and pairs of components not having a large effect. In an exploratory analysis, there was weak anecdotal evidence for a synergistic effect of four components. In the event of uncertain results, calculating BFs can be used to update the strength of evidence of a dataset supplemented with extended recruitment.”*

Abstract: “reminded insensitive but tended” are you able to provide any BF for this statement?;

*The relevant BFs for this conclusion are included in the results section of the abstract and we have added the BF range for the two-way interactive effects: “Data were mainly insensitive but tended to support there being no large main effects of the enhanced version of individual components on consumption (0.22*

*Introduction: It would be helpful to provide some discussion (specific examples) on how Bayes have been used in other domains to provide more insight by means of additional data;*

**We have added further details into the introduction about previous uses of Bayes factors and Bayesian analyses:**

**“To the authors’ knowledge, no DBCIs have used additional data collected to supplement original effectiveness trial findings and no trials have used Bayes Factors to provide further insight based on additional data. However, Bayes Factors have been used in trials for superiority, non-inferiority and equivalence designs to allow for explicit quantification of evidence in favour of the null hypothesis [1]. Bayesian analyses, more generally, are often used in clinical trials for dose finding, efficacy monitoring, toxicity monitoring, and for diagnosis/decision making [2]. For example, Bayesian analyses were used to simultaneously monitor toxicity and efficacy in a parallel phase I/II clinical trial design for combination therapies [3].”**

*Methods – Participants: “were interested in reducing their drinking” how was this measured? Were participants research aware, or were they targeted because they had previously stated an interest in reducing alcohol? Or could it be by downloading DrinkLess they were assumed to be interested in reducing their alcohol consumption?;*

*Methods – Participants: Was a geolocation restriction placed on participants? How can you be sure that users were from the UK?;*

**We have clarified details on the participants in the methods section:**

**“Participants were included in the study if they: were aged 18 or over; lived in the UK (only available on UK Apple app store and users had to select ‘UK’ for ‘Country?’); had an AUDIT score of 8 or above (indicative of excessive drinking); were interested in reducing their drinking (indicated by the question ‘why are you using this app?’ with users choosing ‘interested in drinking less’ over ‘just browsing’); provided an email address and had downloaded a ‘trial version’ of the app (described below).”**

*Methods – Intervention: What were the minor bug fixes, is a summary able to be provided as a supplement?;*

**As the bug fixes are minor, we have included a brief explanation in the manuscript: “The content of the app did not change during the trial except for minor bug fixes (to ensure compatibility with iOS 10).”**

*Results: Results present AUDIT-C score, however this is not discussed previously.*

**We have clarified this in the measures section:**

**“The primary outcome measure was self-reported change in past week alcohol consumption (the difference between one-month follow-up and baseline). Past week alcohol consumption was derived from the frequency (Q1) and quantity (Q2) questions of the AUDIT-Consumption (AUDIT-C) questionnaire.”**

*Results: It would be helpful to have Table 2 represented as supplementary material for those who took part in follow-up;*

**We have added a supplementary table of the participant characteristics for those who responded to follow-up.**

*Results: It would be of interest to discuss further the difference in AUDIT and AUDIT-C score and the role the final two questions (risk taking etc) play;*

**We have discussed the differences between the AUDIT-C and AUDIT scores in the methods section:**

**“The secondary outcome measure was self-reported change in full AUDIT score; in addition to the three questions on consumption in the AUDIT-C, the full AUDIT includes questions assessing harmful alcohol use (e.g. alcohol-related injuries) and symptoms of dependence.”**

*Discussion: “no additional resources were required” – is this the case, was the app provisioned for longer than anticipated?;*

**The app was always intended to be available for the long-term (i.e. not removing it after completion of the trial). We have added more explanation to this section of the discussion:**

**“No additional resources were required to continue data collection within the original trial of Drink Less as the app remained freely available on the UK Apple app store and the notification to complete the follow-up questionnaire had already been programmed.”**

*Discussion: “our decision on which components to retain or remove” – a bit more discussion round this aspect would be helpful to the reader;*

**We have elaborated on this in the discussion:**

**“Analysing the supplemented dataset has allowed us to update our findings and provided more confidence in our original decisions on which components to retain or remove as part of the process of optimising the intervention ^15^ to improve its effectiveness and usability. We are also much clearer that any definitive trial must be powered to detect small effects and designed to inform a pragmatic decision about whether to invest resources in recommending the app. The optimisation of the Drink Less intervention was based on the findings from this study as well as on user feedback and findings from a meta-analysis of the intervention components in digital alcohol interventions associated with effectiveness *[9] *. The findings from this study informed the removal of the ‘Identity Change’ module and retention of the remaining four modules.”**

*Discussion: A 13.2% follow-up rate appears to be very low, do the authors have any reasons for this?;*

**We have included a discussion of this in the limitations:**

**“We acknowledge that the follow-up rate is very low and this is likely to be due to the lack of financial incentive for completing the follow-up survey, which are known to increase response rates in randomised trials (Brueton et al., 2014). Furthermore, the follow-up rate in the extended dataset was lower than for the original trial dataset; this is likely because participants were only contacted via the app for the extended dataset whilst the participants in the original dataset were also contacted via email.”**
